# Supplementary figures and images for: Exposure to Mycobacterium remodels alveolar macrophages and the early innate response to Mycobacterium tuberculosis infection
Source: PLoS Pathog. 2024 Jan 18;20(1):e1011871. doi: 10.1371/journal.ppat.1011871 (PMC10796046; doi:10.1371/journal.ppat.1011871)

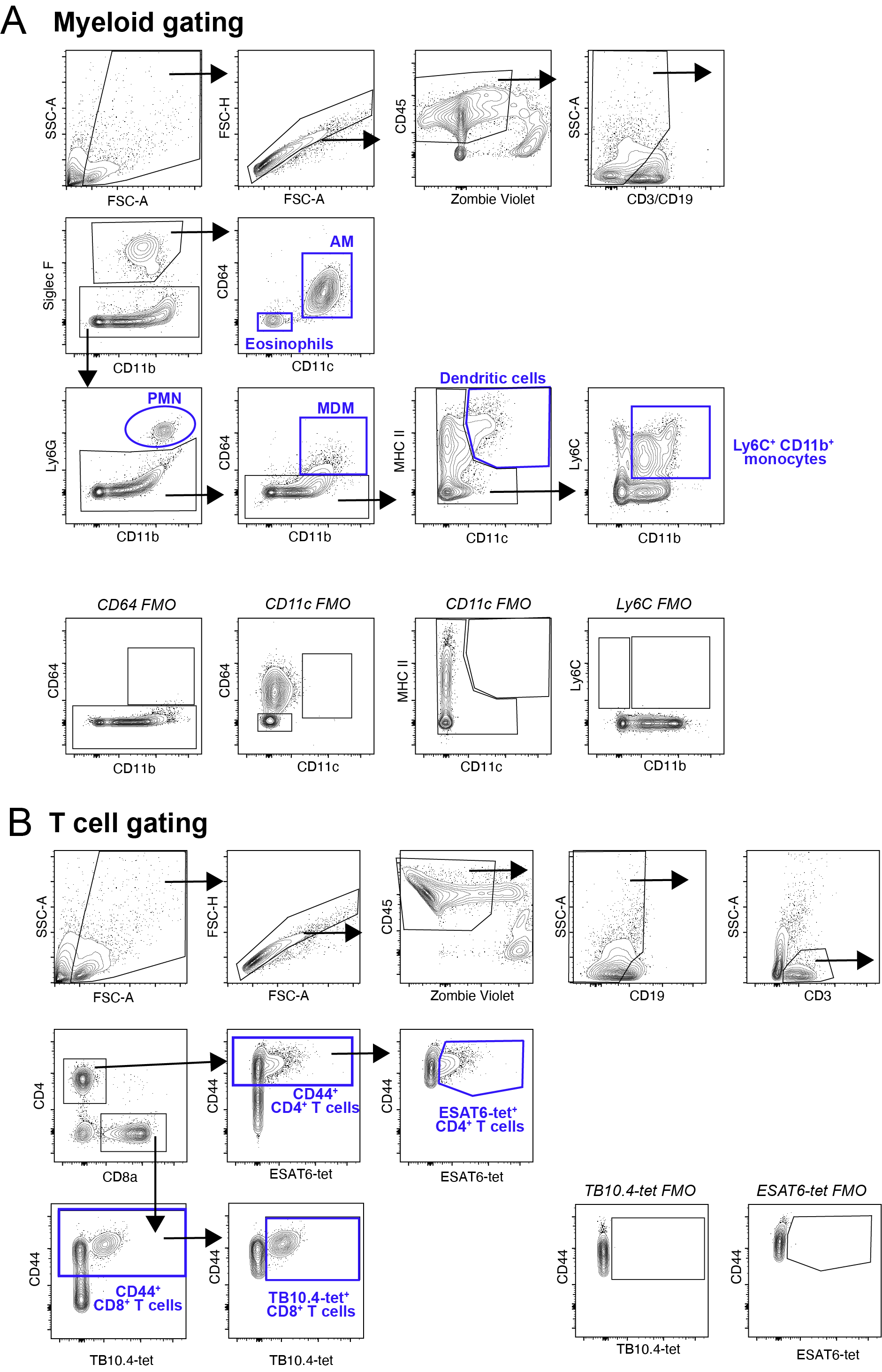

Supplement: S1 Fig — Gating strategies for myeloid (A) and T cell (B) analysis. (TIF) [file ppat.1011871.s001.tif]

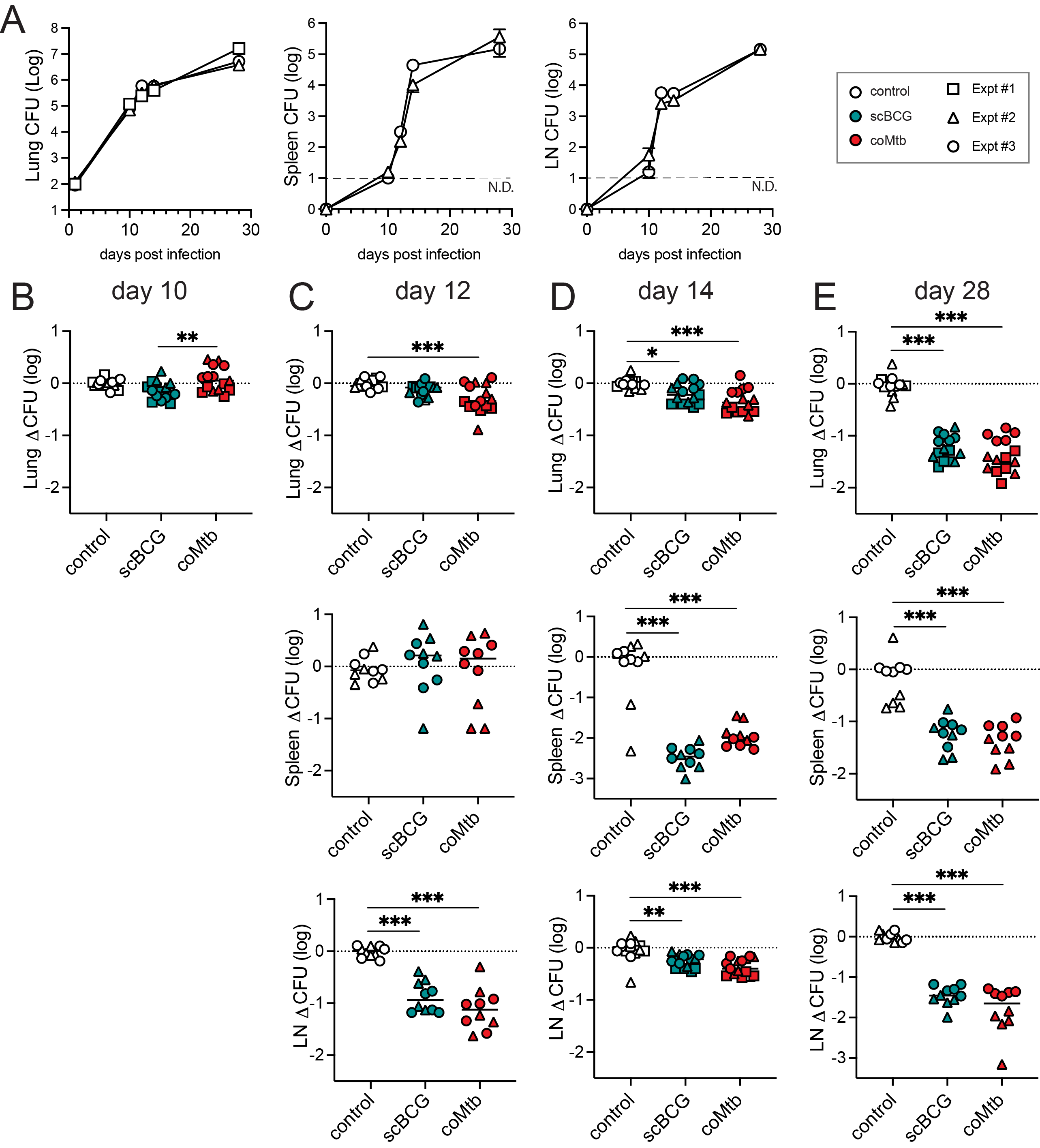

Supplement: S2 Fig — A) Lung, spleen, and lung-draining lymph node (LN) CFU in control mice at deposition, days 10, 12, 14, and 28. B-E) Summary plots of ΔCFU (log) in lung, spleen, and LN following low-dose infection with H37Rv at day 10 (B), day 12 (C), day 14 (D), and day 28 (E). *p < 0.05, **p < 0.01, ***p < 0.001. One-way ANOVA with Tukey post-test. Data compiled from 2–3 independent experiments per condition, with 5 mice per group for each experiment. (TIF) [file ppat.1011871.s002.tif]

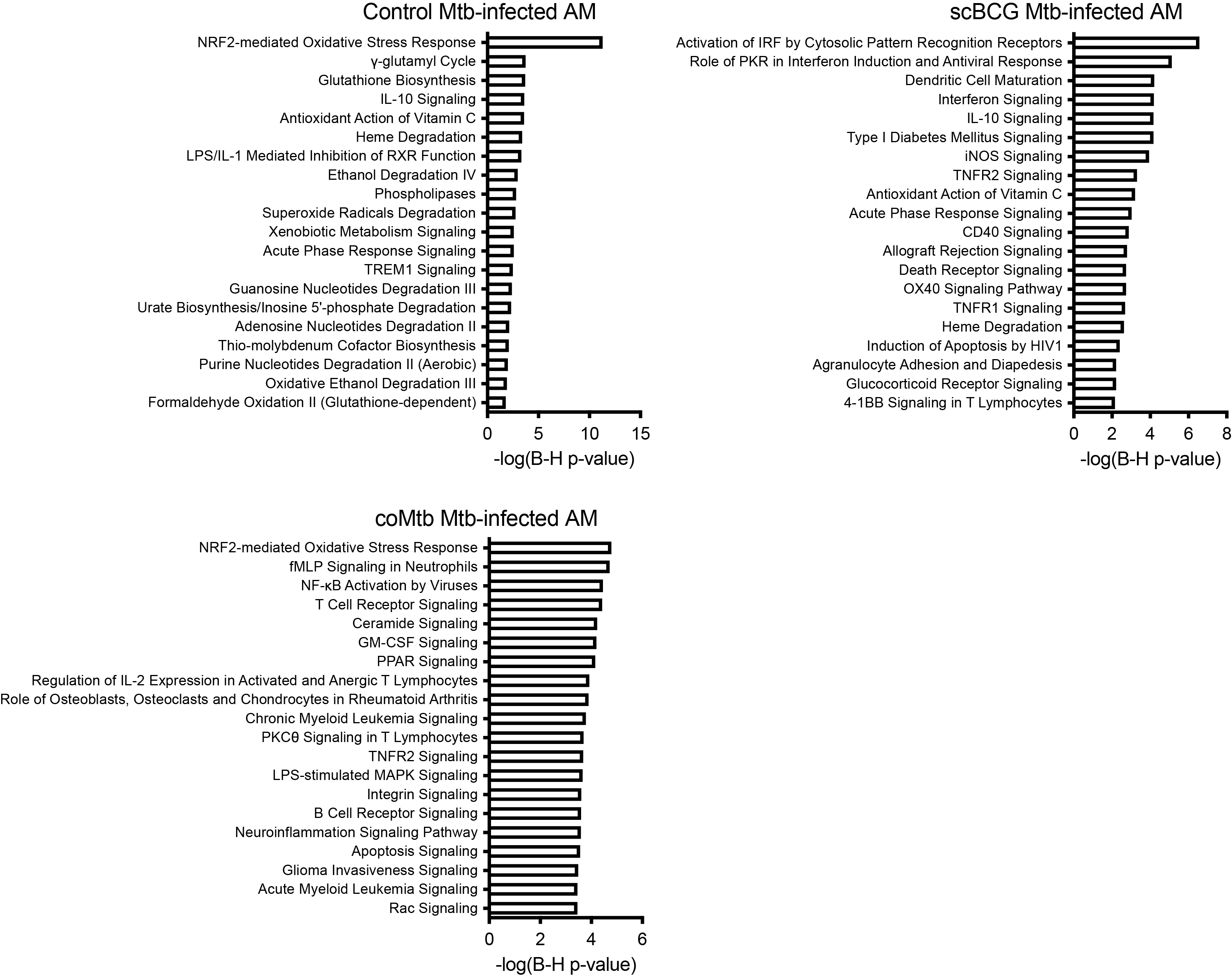

Supplement: S3 Fig — IPA analysis for Mtb-infected AMs from control, scBCG, and coMtb mice 24 hours following high dose mEmerald-H37Rv infection. Data representative of 3 independent experiments per condition. (TIF) [file ppat.1011871.s003.tif]

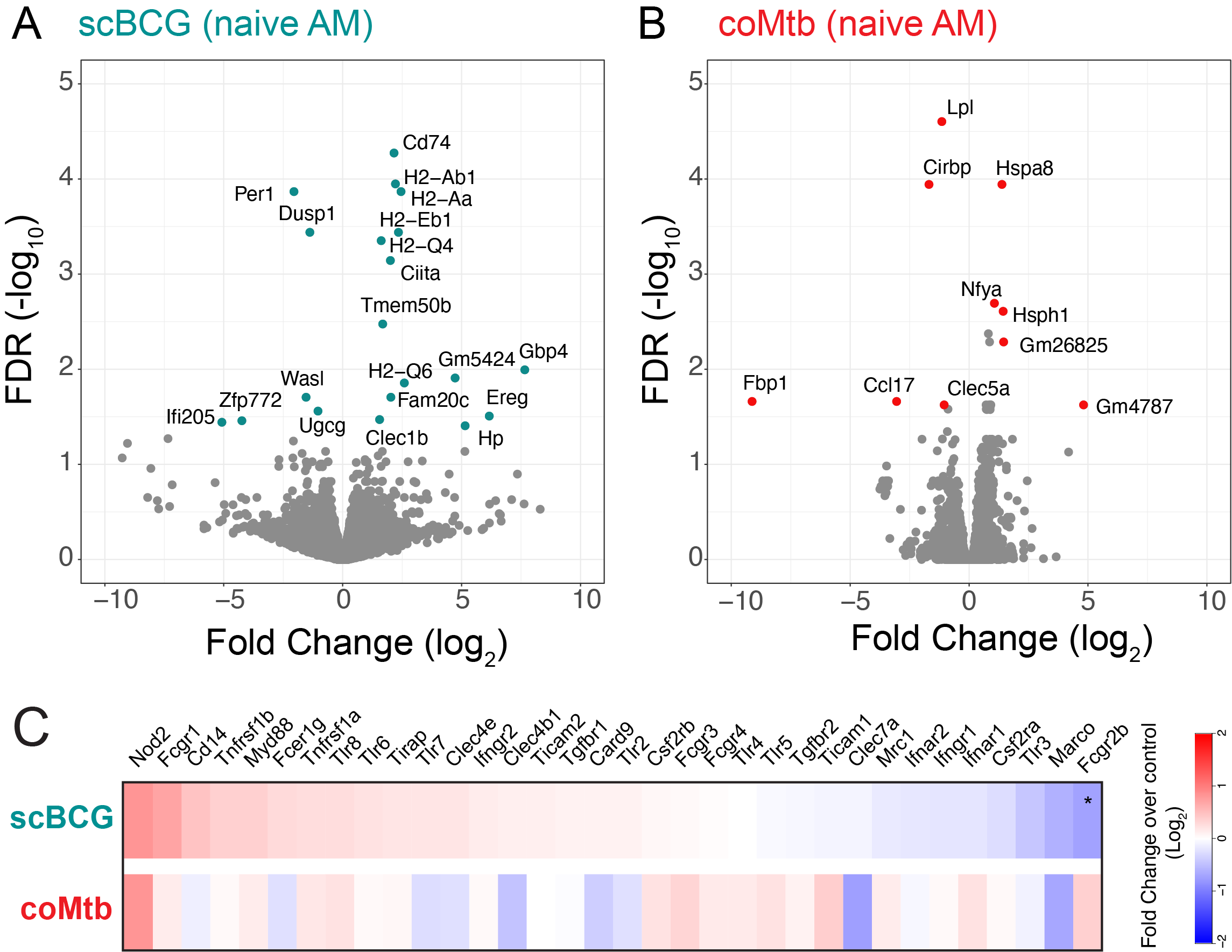

Supplement: S4 Fig — Bulk RNA-seq profiles of naive AMs (isolated alongside Mtb-infected AMs). Gene expression changes within naïve AMs are compared to AMs from control mice: naïve scBCG AM vs naïve control AM; naïve coMtb AM vs naïve control AM. A-B) Volcano plots depicting changes in baseline gene expression of naive AMs from scBCG (A) and coMtb(B) mice compared to naive AMs from control mice. Significantly changed genes (FDR < 0.05, |FC| > 2) highlighted and labeled. C) Gene expression for innate receptors and adaptors of interest, log2 fold change, unstimulated AMs from scBCG and coMtb mice compared to unstimulated AMs from control mice. * FDR < 0.01. Compiled from 2 independent experiments for each condition. (TIF) [file ppat.1011871.s004.tif]

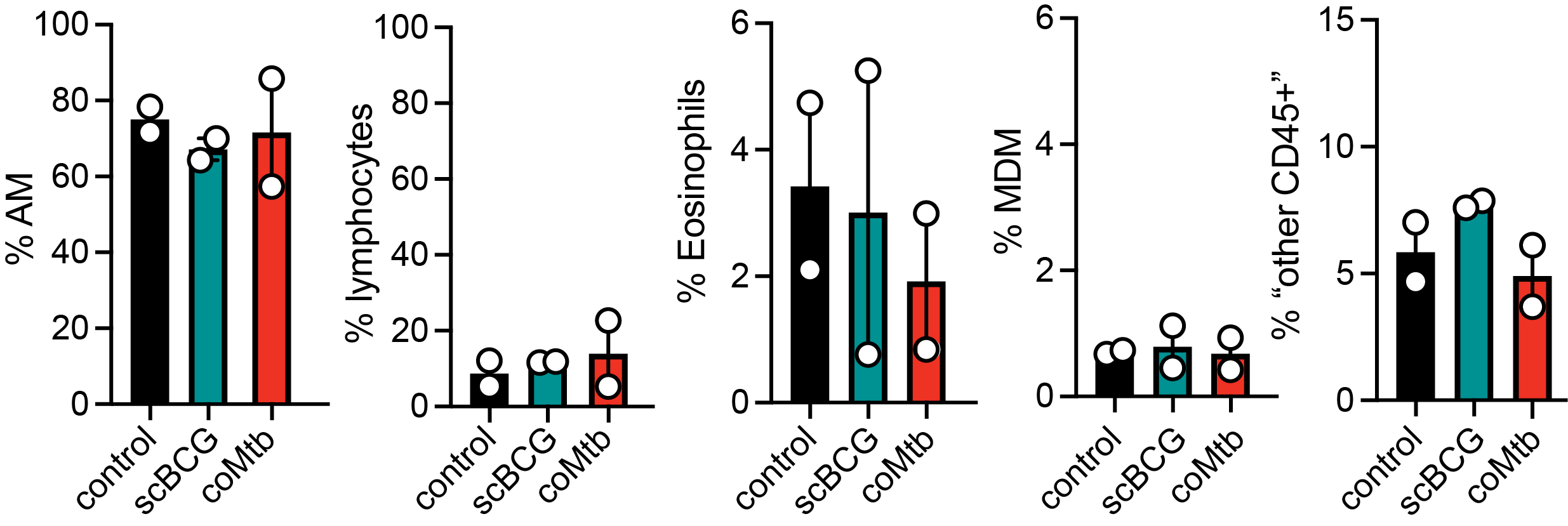

Supplement: S5 Fig — Percentage of each population (AM, lymphocytes, eosinophils, MDM, other CD45+) out of CD45+ ZV-. AM = Siglec F+ CD64+, Eosinophils = Siglec F+ CD64-, lymphocytes = CD3/CD19+, MDM = Siglec F- CD64+, other CD45+ = CD3- CD19- Siglec F- CD64-. Note: One of the two coMtb samples analyzed by flow cytometry did not have an accompanying 10X sample. The second coMtb 10X sample was processed separately without flow analysis. (TIF) [file ppat.1011871.s005.tif]

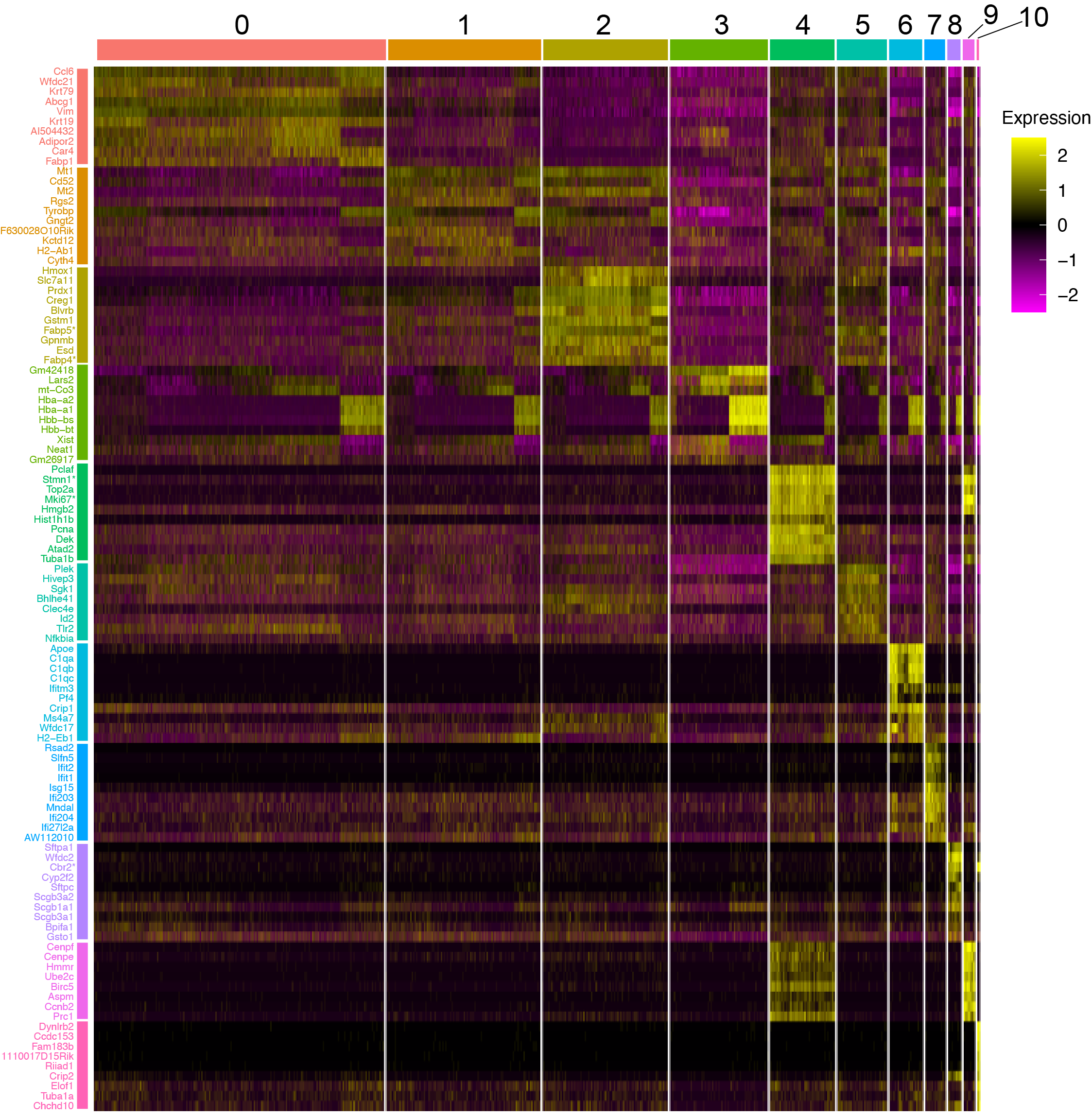

Supplement: S6 Fig — Heatmap of genes that are most differentially expressed for each of 11 clusters with all other clusters. Genes filtered with log fold change threshold of > 0.25 and minimum percentage expression of 25% of cells. All genes but one (Gsto1) had an adjusted p-value of < 1.0x10^-5^. *Five genes (Fabp4, Fabp5, Stmn1, Mki67, Cbr2) met this criterion for more than one cluster, grouped with the more abundant cluster. Data is compiled from two independent experiments, 3 conditions each, for a total of 6 samples. (TIF) [file ppat.1011871.s006.tif]

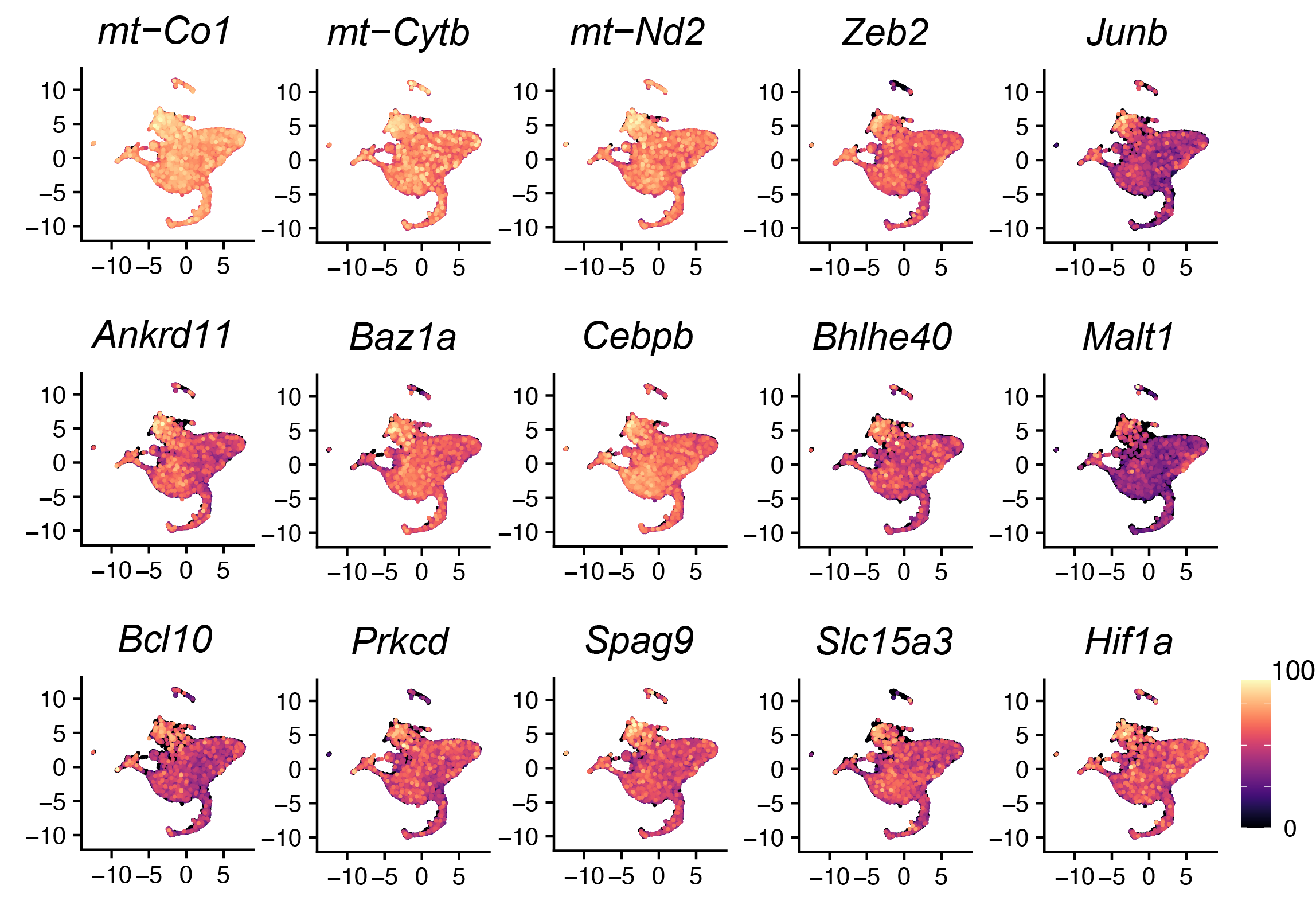

Supplement: S7 Fig — Genes associated with mitochondrial oxidative phosphorylation (mt-Co1, mt-Cytb, mt-Nd2), chromatin remodeling (Ankrd11, Baz1a), macrophage-associated transcription factors (Cebpb, Zeb2, Bhlhe40, Hif1a), and CARD9 signaling (Malt1, Bcl10). Data is compiled from two independent experiments with 3 conditions each, for a total of 6 samples. (TIF) [file ppat.1011871.s007.tif]

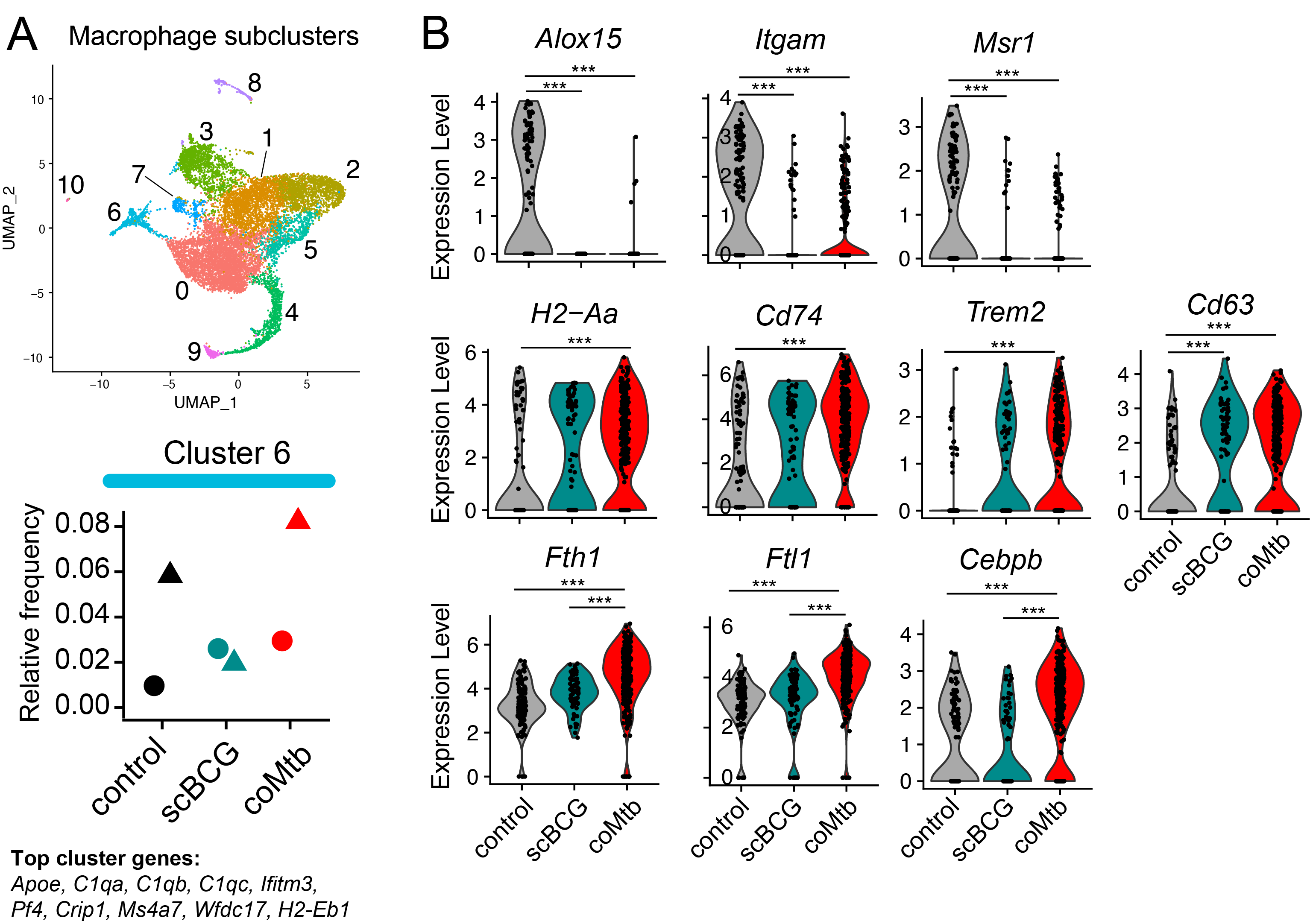

Supplement: S8 Fig — A) Single-cell RNA-sequencing from BAL samples from control, scBCG, and coMtb mice. Subcluster of macrophages with each cluster annotated. Relative frequency of Cluster 6 for each replicate. B) Differentially expressed genes within Cluster 6 between control vs scBCG vs coMtb samples. Wilcoxon Rank Sum Test, Bonferroni adjusted p-value, ***adj-p < 0.001. (TIF) [file ppat.1011871.s008.tif]

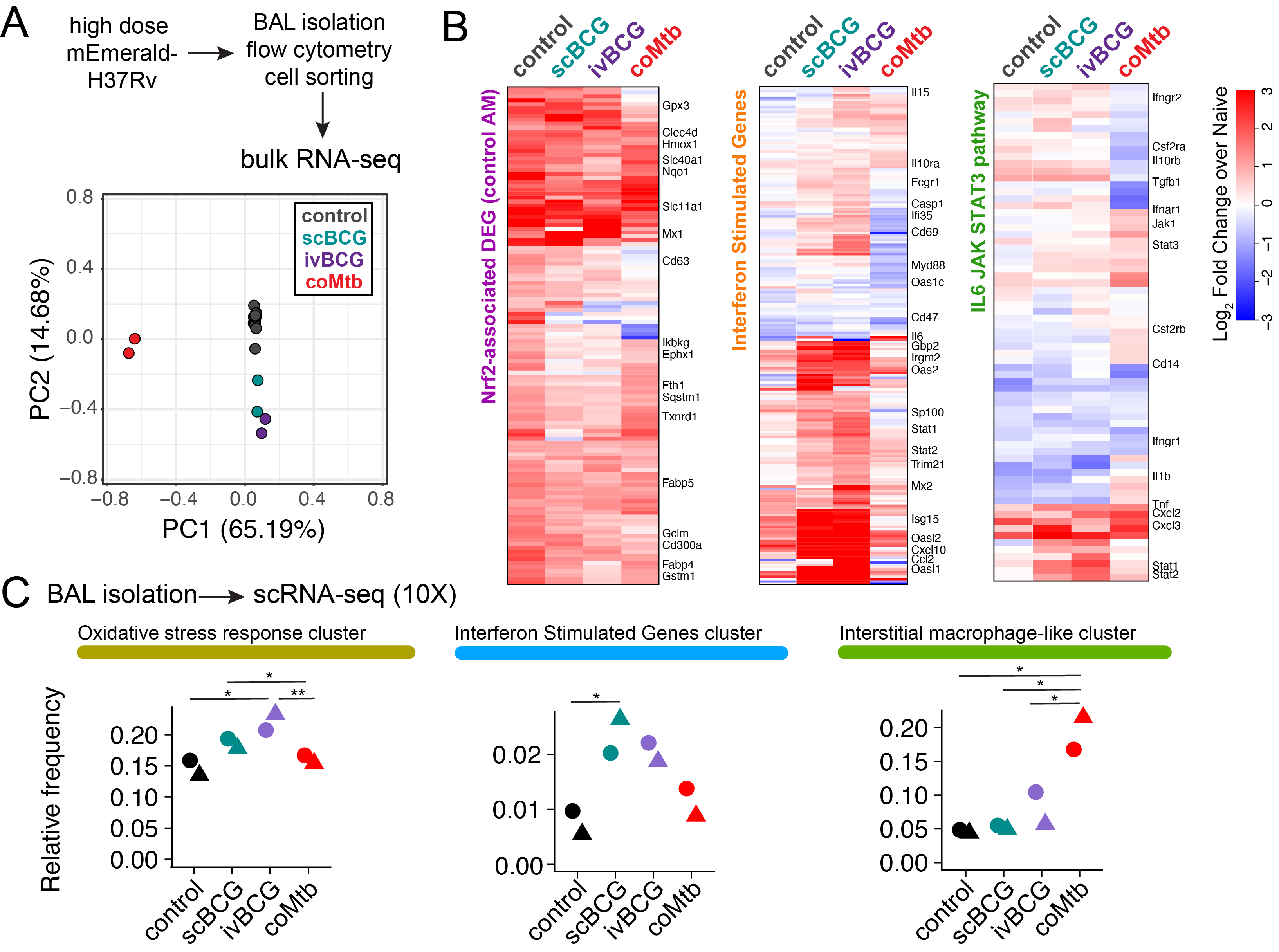

Supplement: S9 Fig — A-B) Bulk RNA-seq gene expression analysis between naive and Mtb-infected AMs 24 hours following high-dose mEmerald-H37Rv infection in mice previously exposed to scBCG, ivBCG, and coMtb, compared to controls. (controls- reported in Rothchild et al, 2019(10); CMTB- reported in Nemeth et al, 2020(25)). A) Principal Component Analysis using DEG (fold change > |2|, FDR < 0.05) in Mtb-infected AMs at 24 hours. B) Heatmap of 131 DEG at 24 hours in Mtb-infected AM (left), Interferon Stimulated Genes, derived from macrophage response to IFNα (fold change >2, p-value < 0.01) Mostafavi et al, 2016 (30)(middle), IL6 JAK STAT3 hallmark pathway (right). C) Relative frequency of 3 key clusters for macrophage subset from control, scBCG, ivBCG, and coMtb scRNAseq BAL samples. (A-B) Compiled from 3+ independent experiments per condition for control, 2 independent experiments per condition for scBCG, ivBCG, and coMtb. (C) Data is compiled from two independent experiments (circle, triangle) with 3 conditions each for a total of 6 samples. (TIF) [file ppat.1011871.s009.tif]

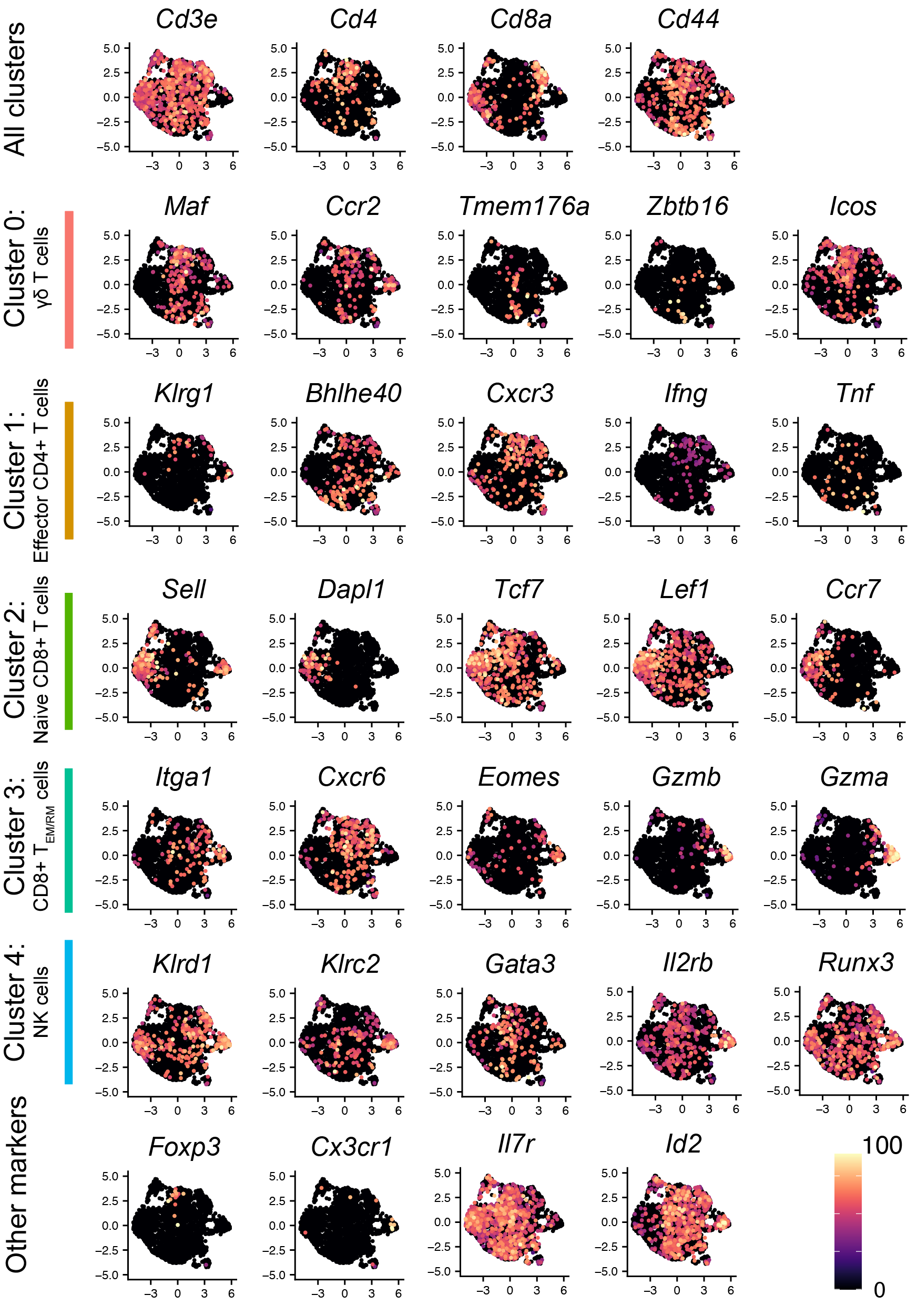

Supplement: S10 Fig — Data is compiled from two independent experiments with 3 conditions each for a total of 6 samples. (TIF) [file ppat.1011871.s010.tif]

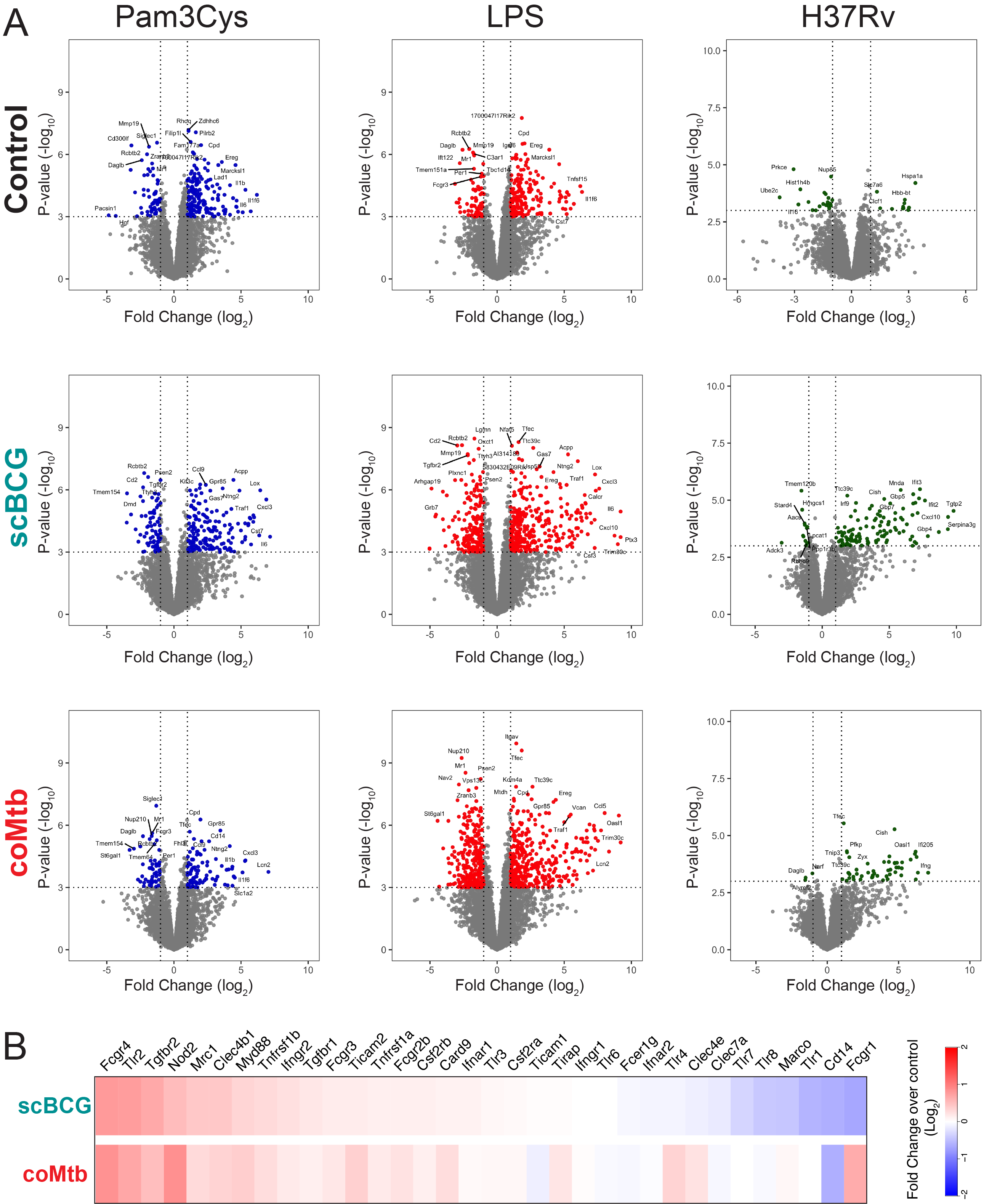

Supplement: S11 Fig — A) Gene expression changes measured by bulk RNA-seq for stimulated AMs compared to respective unstimulated AMs (i.e., LPS-stim control AM vs unstim control AM; LPS-stim scBCG AM vs unstim scBCG AM; LPS-stim coMtb AM vs unstim coMtb AM). AMs were stimulated for 6 hours with Pam3Cys (10 ng/ml), LPS (10 ng/ml), or H37Rv (effective MOI ~2:1). Volcano plots depict fold change (log2) and P-value (-log10) for each stimulation condition for each of the three groups (control scBCG, coMtb) compared to the respective unstimulated controls. DEG (p-value < 0.001; |fold change| > 2) highlighted and labeled, space permitting. B) Baseline gene expression for innate receptors and adaptors of interest from scBCG and coMtb AM compared to control AM, log2 fold change, unstim scBCG AM vs unstim control AM; unstim coMtb AM vs unstim control AM. Compiled from 3 independent experiments. (TIF) [file ppat.1011871.s011.tif]

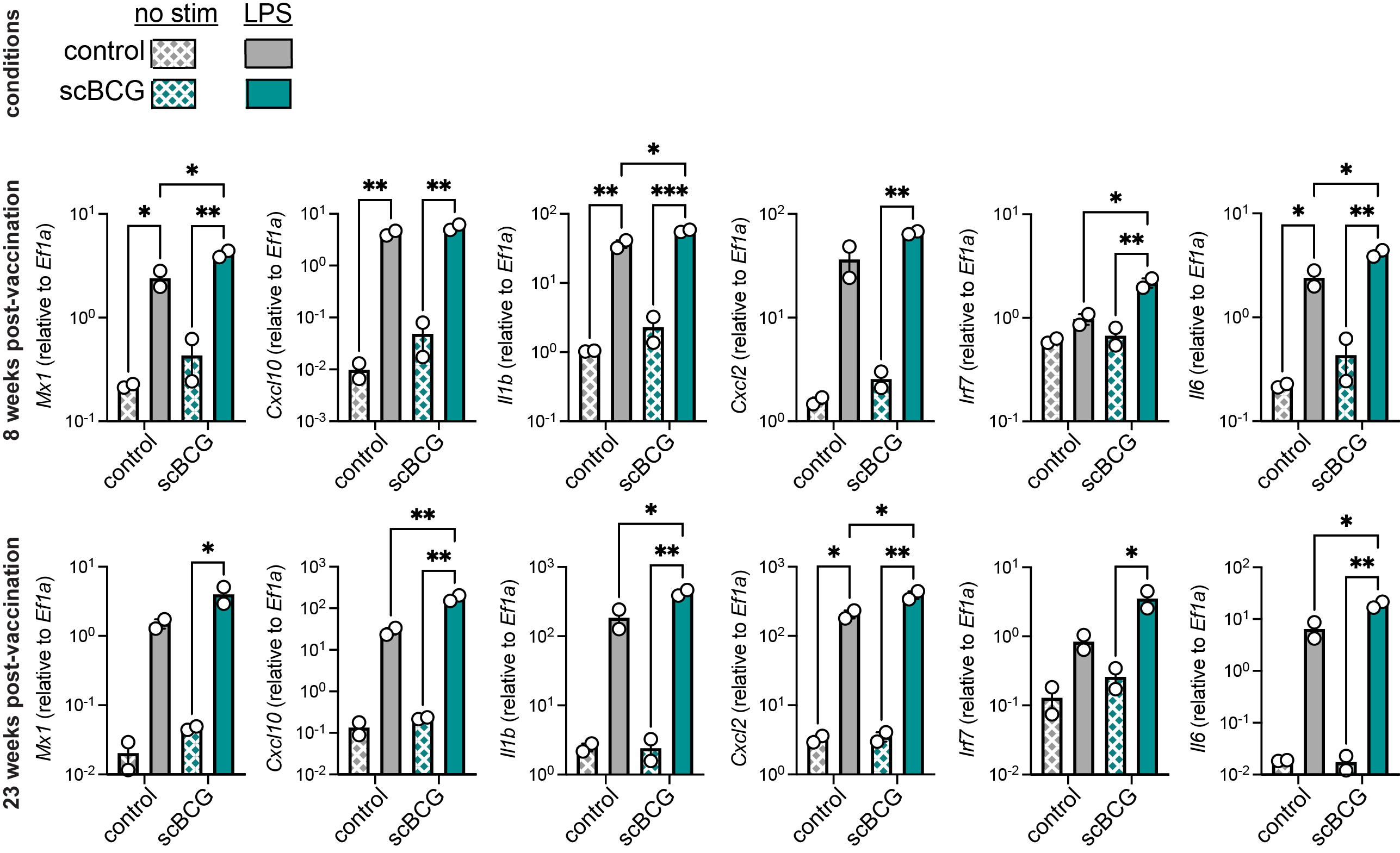

Supplement: S12 Fig — Gene expression of Mx1, Cxcl10, Il1b, Cxcl2, Irf7, and Il6 as measured by qPCR in AMs isolated by BAL from mice 8 and 23 weeks following scBCG vaccination and from age-matched controls, with and without LPS (10 ng/ml) stimulation. Data is representative of technical AM duplicates from a single experiment. (TIF) [file ppat.1011871.s012.tif]
